# Virtual Reality for Developing Patient-Facing Communication Skills in Medical and Graduate Education: Protocol for a Scoping Review

**DOI:** 10.2196/53901

**Published:** 2024-02-01

**Authors:** Nairy Khodabakhshian, Kyla Gaeul Lee, Tulip Marawi, Maryam Sorkhou, Sobiga Vyravanathan, Nicole Harnett

**Affiliations:** 1 Institute of Medical Science University of Toronto Toronto, ON Canada; 2 Labatt Family Heart Centre The Hospital for Sick Children Toronto, ON Canada; 3 Department of Psychiatry Sinai Health Toronto, ON Canada; 4 Sunnybrook Research Institute Toronto, ON Canada; 5 Addictions Division Centre for Addiction and Mental Health Toronto, ON Canada; 6 Department of Radiation Oncology University of Toronto Toronto, ON Canada

**Keywords:** communication, medical education, patient-facing, scoping review, technology, virtual reality

## Abstract

**Background:**

Clinician-patient communication is an integral component in providing quality medical care. However, research on clinician-patient communication has shown overall patient discontent with provider communication skills. While virtual reality (VR) is readily used for procedural-based learning in medical education, its potential for teaching patient-facing communication skills remains unexplored. This scoping review aims to evaluate the effectiveness and feasibility of VR applications used for patient-facing communication skills development in medical education.

**Objective:**

The primary objective is to synthesize and evaluate the effectiveness of available VR tools and applications used for patient-facing communication skills development in medical education. The secondary objectives are to (1) assess the feasibility of adapting VR applications to develop patient-facing communication skills in medical education and (2) provide an overview of the challenges associated with adapting VR applications to develop patient-facing communication skills in medical education.

**Methods:**

A total of 4 electronic databases (ERIC, Embase, PubMed, and MEDLINE) were searched for primary peer-reviewed articles published through April 11, 2023. Articles evaluating the implementation of non-, semi-, and fully immersive VR training for patient- or caregiver-facing communication skills training provided to graduate, medical, or other allied health care professions students were included. Studies that assessed augmented reality, mixed reality, artificial intelligence, or VR for non–communication-based training were excluded. Study selection will include a title, abstract, and full-text screening by 4 authors. Data from eligible studies will be extracted and entered into a database and presented in tabular format. Findings will be reported according to the PRISMA (Preferred Reporting Items for Systematic Reviews and Meta-Analyses) guidelines for scoping reviews.

**Results:**

As of April 11, 2023, the search strategy has been confirmed and the search has been completed. We are currently at the title and abstract screening stage. Once complete, the articles will undergo full-text screening according to eligibility criteria as described in the methods.

**Conclusions:**

The findings of this review will inform the development of a graduate-level clinical skills research course within the Institute of Medical Science graduate department at the University of Toronto. It is also expected that these findings will be of interest to other health care–specific faculties inside and beyond our institution. Further, our scoping review will summarize the limited field of literature on VR use in medical communications training and identify areas for future inquiry.

**International Registered Report Identifier (IRRID):**

DERR1-10.2196/53901

## Introduction

Effective communication skills are crucial for health care professionals to establish trust and build rapport with their patients, facilitate shared decision-making, and deliver high-quality care. Poor communication between clinicians and patients has been associated with decreased health care quality, increased human and economic cost of care, disenrollment from health care plans, poor adherence to recommended treatments, and propensity to sue for medical malpractice [1-4]. Accordingly, American medical colleges [5] and Canadian medical schools [6] have stated that clinician-patient communication is an integral component of quality medical care and highlighted the need for formal training programs at the undergraduate, postgraduate, and continuing education levels. However, research on clinician-patient communication has shown overall patient discontent even when clinicians indicate their own communication to be good or excellent [7]. Although there has been increasing emphasis placed on communication skills training in most health care curricula, significant challenges remain in their implementation and evaluation. These training approaches are often limited in time, resources, personnel, contextualization, unclear frameworks, and teaching strategies [8]. Incorporating educational tools that promote and develop patient-focused communication skills is imperative for the delivery of comprehensive care by health care professionals.

Virtual reality (VR) has emerged as a promising tool that addresses the challenges associated with limited access to health care institutions, personnel, contextualization, and feedback to evaluate frameworks and teaching outcomes. VR is defined as an educational tool that uses computer technology to generate a 3D image or environment with which a user can interact in a seemingly real or physical manner [9]. Presently, VR has been mainly used for training in procedural skills, typically for surgical training, but has been expanding to other applications as well [10]. There are currently 3 main categories of VR: nonimmersive, semi-immersive, and fully immersive. Nonimmersive VR is typically a screen-based display that is connected to handheld mechanical or haptic units [11]. Nonimmersive VR is commonly used to develop technical psychomotor skills, such as those needed in endoscopic surgery [12]. In semi-immersive VR, users use a VR headset and dedicated controllers to interact with a 3D VR, usually spanning 180° [13]. The addition of body sensors provides a fully immersive experience in which a user is placed entirely in a virtual environment, and their awareness of the real world is disconnected [9]. Semi- and fully immersive VR is particularly useful for teaching appropriate responses to stressful scenarios such as mass casualties, emergency surgical procedures, and cardiopulmonary resuscitation [14,15].

VR offers a simulated environment for diverse applications that enable learners to practice various skills with patients and caregivers in a safe and controlled setting without relying on health care facilities or personnel [10]. For example, Izard et al [16] demonstrated the effective use of VR in teaching surgical trainees detailed practical knowledge about surgical procedures. Another study by Birrenbach et al [17] leveraged VR during the COVID-19 pandemic to explore the short- and long-term effectiveness of VR simulation versus traditional learning methods for training health care professionals in hand disinfection, nasopharyngeal swab-taking, and donning or doffing of personal protective equipment [17]. VR technology can also provide learners with immediate feedback on their communication skills, tangible learning outcomes for educators, and allow for the repeated practice of challenging scenarios that may not be possible in real-life clinical settings [11,12]. Zackoff et al [14] used VR to train medical students in managing respiratory distress. The participants reported the VR environment as equal or superior to the perceived effectiveness of other training modalities such as standardized patients and high-fidelity mannequins. The learners also perceived VR as equally effective to standardized patients for communication training [14].

While less researched compared to technical medical skills, the rapid progression of technology has expanded the possibilities of using VR as a training tool targeting communication skills. Immersive first-person VR was found to be successful in teaching effective health dialogues and communication skills. For example, clinicians and postgraduate medical residents who used VR were more confident in and more capable of communicating difficult medical information to patients [18]. Furthermore, undergraduate research students who were trained with VR were better prepared for real-world patient interactions that involved obtaining consent [19]. Overall, VR was able to improve the dialogue performance of students with little to no previous communication training, with students posttraining being able to respond to patient questions in a more accurate and timely manner [19]. In another study, VR allowed participants to replay their experiences to help them recognize and analyze their interactions and emotions to help inform their future communications with patients [20]. Altogether, VR delivers immersion and realism, reflecting real-world scenarios that increase empathetic communication with patients [21-23]. Despite the strong evidence presented in these studies, there is still a lack of consensus on the most effective VR tools and applications for patient-facing communication skills development in medical or health care professional education (referred to as “medical education” hereafter).

Effective communication skills are crucial for health care professionals to deliver high-quality care and establish positive patient-clinician relationships, ultimately improving patient outcomes. The use of VR technology for communication skills training in medical education has the potential to provide learners with a safe and controlled environment to practice challenging scenarios and receive immediate feedback on their communication skills. The majority of VR participants agreed that the technology was reflective of real life, strongly suggesting VR as a capable educational tool [21,23]. VR was also a highly rated learning experience and preferred over standard didactic lectures [24,25]. However, the applications of VR for communication skills development in health care professionals remain limited. This scoping review aims to synthesize and evaluate the available evidence on the effectiveness of VR tools and applications for patient-facing communication skills development in medical education and inform educators and health care professionals on the potential benefits and limitations of this technology.

## Methods

### Approach

To inform our objectives, we will conduct a scoping review following the methodology described by Arksey and O’Malley [26] and Levac and colleagues [27]. As our research aims to describe the application of an emerging tool (VR) in medical education and we intend on applying the findings of the scoping review to the development of a new clinical research skills course, a scoping review is an appropriate approach. While similar in rigor to systematic reviews, a scoping review allows for a preliminary assessment of the size of existing evidence to inform future directions for research priorities by identifying knowledge gaps, usually in an area of ongoing research and knowledge synthesis [28,29].

The scoping review approach encompasses the following six stages: (1) identifying the research question; (2) identifying relevant studies; (3) selecting studies; (4) charting the data; (5) collating, summarizing, and reporting the results; and (6) engaging in knowledge translation and stakeholder discussions. We will adhere to the PRISMA-ScR (Preferred Reporting Items for Systematic Reviews and Meta-Analyses extension for Scoping Reviews) [30].

#### Stage 1: Identifying the Research Question

The objective of this scoping review is to outline and assess the available VR tools and applications used for patient-facing communication skills development within the context of medical education. To meet this objective, we have developed the following research questions:

1. How is VR implemented in a medical education setting to develop patient-facing communication skills?
2. Is VR an effective tool to develop patient-facing communication skills in a medical education context?
3. Is adapting VR tools to develop patient-facing communication skills in medical education feasible?
4. What are the challenges associated with adapting VR tools and applications to develop patient-facing communication skills in medical education?

#### Stage 2: Identifying Relevant Studies

To inform our study selection, we developed an operational definition of VR, communication, and medical education students.

##### Operational Definition of VR

We defined VR as a computer-based tool that generates a 3D image or environment that allows the participant to look about and navigate within a seemingly real or physical world [21,31,32]. Key attributes of VR in our definition included the use of (1) 3D imaging, (2) the ability to actively interact with the virtual environment, and (3) visual and auditory feedback that allows the user to feel immersed in the virtual environment [33]. In order to capture the full breadth of VR, our operational definition included nonimmersive (eg, screen-based VR simulators), semi-immersive (ie, immersive VR without physical movement), and fully immersive VR (usually using head-mounted displays and hand-held equipment) [31].

##### Operational Definition of Communication

We define communication as effectively engaging in conversation and exchanging information with patients, caregivers, or decision-makers regarding a medical condition in simple, clear, and plain language. Within the context of trainees in a medical education setting, effective communication skills include, but are not limited to (1) obtaining informed consent to perform a procedure; (2) engaging in difficult conversations (eg, breaking bad news); (3) collecting personal, sensitive, and confidential patient information in an ethical manner; (4) listening attentively to patient or caregiver concerns to assess the patient’s health and condition; (5) maintaining professional relationships with patients and caregivers; and (6) validating and addressing patient or caregiver emotions and concerns [34,35].

##### Operational Definition of Medical Education Students

For our scoping review, we identified medical education students as any student in a medical or health care profession field. These students included but were not limited to, graduate, medical, nursing, physical therapy, physiotherapy, occupational therapy, or other allied health care professions students that are required to interact directly with patient populations in the delivery of their care and would thus benefit in communications training in their work. All students and trainees that may be included in the circle of care, either directly or indirectly, that communicated with patients were included in our definition of medical education students.

### Search Strategy

In collaboration with a health sciences research librarian at the University of Toronto, a member of our team (MS) developed the search strategy to locate published and unpublished studies on PubMed, ERIC, Embase, and MEDLINE databases on April 11, 2023. The search was limited to data that were published from January 2000 to April 2023.

The search strategy explores specific search terms within subject headings, titles, abstracts, and keywords (Textbox 1). The search strategy, including all identified keywords and index terms, was adapted for each database to account for appropriate MeSH (Medical Subject Headings) terms. Textbox 1 shows the search strategy used for each database, with concepts combined with Boolean operators AND and OR. Further potentially relevant studies will be identified by conducting a search of the references of included articles and relevant systematic reviews and meta-analyses.

**The search strategy used to obtain studies.**

1. computer simulation/ or augmented reality/ or virtual reality/
2. computer-assisted instruction/ or simulation training/ or high fidelity simulation training/ or patient simulation/
3. ((virtual or mixed or augment*) adj3 (realit* or simulation*)).tw,kf.
4. (simulation* adj3 (train or trained or training* or patient* or instruction*)).tw,kf.
5. ((computer* or computational) adj3 (model* or simulation* or assisted instruction)).tw,kf.
6. 1 or 2 or 3 or 4 or 5
7. (class* or postsecondary or educat* or instruct*).tw,kf.
8. 6 and 7
9. ((clinic* or patient or medic* or communicat*) adj2 (skill* or interact* or instruct*)).tw,kf.
10. 8 and 9

#### Stage 3: Study Selection

##### Overview

The study selection phase will include the following two screening phases: (1) title and abstract screening and (2) full-text screening. First, 4 authors (NK, KGL, TM, and MS) will screen the titles and abstracts of all eligible studies identified through searching the electronic databases for relevance according to the inclusion and exclusion criteria outlined in Textbox 2. Each title and abstract will be screened by 2 of 4 authors (NK, KGL, TM, and MS), and in order for an article to move past the first screening phase, it must be accepted for inclusion by 2 authors. Disagreements will be resolved through a discussion among at least 3 of the following 4 authors: NK, KGL, TM, and MS. Second, these 4 authors (NK, KGL, TM, and MS) will download and review the full text of all articles passing the first screening phase. Similar to the first screening phase, each article will be reviewed by 2 of 4 authors (NK, KGL, TM, and MS), and in order for an article to move past this screening phase, it must also be accepted for inclusion by 2 authors. Disagreements will be resolved through a discussion among all authors. Screening and study selection will be conducted using the Covidence reference management system.

**Study inclusion and exclusion criteria used during screening.**

**Inclusion criteria**

- Published in a peer-reviewed journal in the English language.
- Participants are graduate medical, nursing, physical therapy, physiotherapy, occupational therapy, or other allied health care professions students receiving patient-facing communication skills training.
- Study evaluates the implementation of nonimmersive, semi-immersive, or fully immersive virtual reality training for patient- or caregiver-facing communication skills training.
- Study reports (quantitatively or qualitatively) the learning outcomes of students, the teaching outcomes of educators, or educators’ and learners’ perspectives on virtual reality content and delivery.
- Published after 2000.

**Exclusion criteria**

- Commentaries, editorial notes, systematic reviews, meta-analyses, opinion articles, protocols, dissertations, or book chapters.
- Studies using any form of virtual reality for non–communication-based clinical skills (eg, surgical planning and therapeutic or treatment applications).
- Studies using augmented reality, mixed reality, artificial intelligence, or robotics.
- Studies using manikins for simulation training.

##### Inclusion Criteria

Only articles published in the English language will be considered for this scoping review. Only articles that are peer-reviewed will be considered in order to ensure that the quality of the articles reviewed is of the highest standard. Articles published only after the year 2000 will be included, as the use of technology in education only became more widespread in the early 2000s. Although most of the literature on VR in medical education was published after 2010, there are relevant articles that were published between 2000 and 2009, as 3 of the 9 studies included in the systematic review by Rourke [36] were published between 2000 and 2009. Similarly, 2 other systematic reviews [37,38] included studies published between 2000 and 2009. We adhered to the PICO framework to define the populations, interventions, comparisons, outcomes, and study designs eligible for our review. Studies were included if they met the following criteria: studies including undergraduate, graduate, medical students, medical residents, and any health care professional (ie, population); studies assessing either asynchronous or synchronous VR training methodologies specifically designed for the cultivation of clinical skills in medical education (ie, intervention); studies may or may not comprise of a control group of learners who do not receive VR training (ie, comparison); and studies that assess either the learning outcomes, feasibility, efficacy, and impact of the VR technology in relation to clinical skills teaching methodologies (ie, outcomes).

##### Exclusion Criteria

We aim to further develop a clinical research skills graduate-level course focused on patient-facing communication skills. Therefore, we excluded articles that outline the utility of VR for non–communication-based clinical skills, such as studies using VR to teach surgical procedures, other field-specific medical interventions, combining and administering pharmacotherapy, pain management, and other noncommunication skills or practices. We additionally excluded commentaries, editorials, other reviews and meta-analyses, opinion articles, protocols, dissertations, conference abstracts, and book chapters. Studies in a language other than English were excluded, in addition to studies using non-VR tools to teach clinical communication skills, such as manikins, CD-ROMs, or roleplaying actors. As our aim is to inform the development of a graduate-level clinical research skills course using VR, we restricted our search to only capture VR applications and excluded other forms of experiences, including augmented reality (AR) and mixed reality (MR). While VR allows the user to be fully immersed in the virtual environment, AR and MR supplement and integrate VR with the user’s reality [39,40]. As our course intends to use technology to simulate patient-facing scenarios to teach medical communications skills to students, AR and MR were not considered due to the low fidelity of these technologies. Textbox 2 presents a comprehensive overview of our review’s inclusion and exclusion criteria.

#### Stage 4: Charting the Data

Data from studies meeting the inclusion criteria will be extracted and entered into a database created by the study team. Proposed data fields for extraction include: bibliographic information (ie, author, year of publication, and study’s PubMed ID), location of publication, reported time frame, type of study, participant population (ie, student field of study, student year of study, and affiliated institution), description of the VR tool (eg, nonimmersive, semi-immersive, and fully immersive), technology used to deliver the VR (eg, computer screen and head-mounted device), reported student experience (eg, satisfaction and ease of use), developer and instructor experience (eg, lessons learned), reported barriers and facilitators to the use of VR (if available), and communication-related outcomes.

This developed database will be revised upon piloting the data extraction of 25 articles to ensure that the data relevant to the aforementioned aims are satisfied.

#### Stage 5: Collating, Summarizing, and Reporting Results

The aim of our scoping review is to synthesize the evidence describing the effectiveness and impact of and lessons learned in the feasibility and implementation of VR applications and tools used for patient-facing communication skills. Accordingly, we will use a narrative-qualitative approach to synthesis. Descriptive data about the included studies (ie, location of study, type of study, and participant population) will be reported. Collated data pertaining to effectiveness, impact, lessons learned, and facilitators and barriers to implementation and use of VR will be organized into key themes, then presented through narratives and tables. The results of the search will be included in the final scoping review and presented as a PRISMA (Preferred Reporting Items for Systematic Reviews and Meta-Analyses) flow diagram. Data will be charted using a structured form and narratively summarized. Additionally, we will identify and report gaps in the available literature. Lastly, the review’s findings will be considered within a broader context of research, practice, and educational design implications.

#### Stage 6: Stakeholder Discussions

The findings from this scoping review will help modify the clinical research skills graduate course that is being developed by the authors for the Institute of Medical Science (IMS) graduate program. The findings will be shared with the IMS Curriculum Committee, and the recommendations from this committee will be reviewed to further develop the course, which will undergo the governance process at the University of Toronto. The IMS would like to offer this course to students from different clinical departments in the future, and having this review include participants who are graduate medical, nursing, physical therapy, physiotherapy, occupational therapy, or other allied health care professions students will help inform course development.

### Ethical Considerations

No protocol was registered, and research ethics approval was not sought as all data are publicly available.

## Results

As of April 11, 2023, the search strategy has been confirmed, and the search has been completed. After removing duplicates, our search identified 4141 studies eligible for title and abstract screening. Of these studies, 89 were included for full-text screening. Currently, 4 authors (NK, TM, KGL, and MS) are completing the full-text screening. We anticipate that full-text screening will be completed by February 2024. Following this stage, the results of the review will be tabulated, visualized, and summarized descriptively. The target date for manuscript submission is June 2024.

## Discussion

### Principal Results

VR is emerging as a promising educational tool in medical education that can address the challenges associated with limited resources, cost, and the provision of immediate feedback. This scoping review aims to synthesize and assess the effectiveness of available VR tools and applications to teach patient-facing communication skills in medical education. In addition, this review will assess the feasibility of implementing VR applications and provide an overview of the challenges and lessons learned in VR application implementation and use in medical education.

### Implications and Conclusions

Immediately, the findings of this review will inform the development of a graduate-level VR-based clinical skills research course within the IMS at the University of Toronto, but it is expected that these findings will be of interest to other health care–specific departments within and beyond our institution and will guide future research questions. For example, VR use in medical education has largely focused on technical skills in, for example, anatomy or surgery to date [41,42], with limited literature on VR use in medical communications or professional skills training [10]. Nevertheless, of the scarce literature available on communications and professional skills training in medical education, VR has been shown to foster the development of various competencies, including interprofessional collaboration [43,44], empathy and compassion [45,46], and confidence [47]. Moreover, given the significance of communication as a crucial skill in various medical education specialties, our scoping review holds relevance for educational settings across all medical specialties wherein clinicians and researchers engage with patients and caregivers. Therefore, our scoping review will contribute to this growing field by summarizing the limited field of literature on VR use in medical communications training, informing educators and health care professionals of the potential benefits and limitations of this technology, and identifying areas for future inquiry.

### Limitations

Our proposed protocol has potential methodological limitations and limitations related to the current use of VR in medical education. We restricted our search to only include VR applications and excluded AR or MR applications; therefore, our review is not equipped to draw any conclusions regarding AR or MR use in medical communication skills training. Moreover, we only included studies reported in the English language, and therefore we may potentially not entirely capture findings reported in other languages. Additionally, we recognize the current technological and accessibility limitations in the utility of VR within medical education. For example, implementing VR applications requires costly resources, including specialized hardware of high quality and the expertise of trained personnel, which could present technological barriers and create disparities in accessing the technology among academic institutions [48]. From a pedagogical perspective, previous authors have outlined limitations, including decreased face-to-face communication, the need for robust and adequate evaluation procedures that assess real-life skills, and the importance of more research establishing VR as an effective education for clinical and communication skills [49]. However, a comprehensive overview of the limitations of VR is beyond the scope of this protocol and has been the focus of other reviews [48,49].

## Abbreviations

AR: augmented reality
IMS: Institute of Medical Science
MeSH: Medical Subject Headings
MR: mixed reality
PICO: populations, interventions, comparisons, and outcomes
PRISMA: Preferred Reporting Items for Systematic Reviews and Meta-Analyses
PRISMA-ScR: Preferred Reporting Items for Systematic Reviews and Meta-Analyses extension for Scoping Review
VR: virtual reality
